# Curcumin Improves Amyloid β-Peptide (1-42) Induced Spatial Memory Deficits through BDNF-ERK Signaling Pathway

**DOI:** 10.1371/journal.pone.0131525

**Published:** 2015-06-26

**Authors:** Lu Zhang, Yu Fang, Yuming Xu, Yajun Lian, Nanchang Xie, Tianwen Wu, Haifeng Zhang, Limin Sun, Ruifang Zhang, Zhenhua Wang

**Affiliations:** 1 Key-Disciplines Laboratory Clinical-Medicine of Henan, Zhengzhou, Henan, PR China; 2 Department of Neurology, First Affiliated Hospital of Zhengzhou University, Zhengzhou, Henan, PR China; 3 Department of Intensive Care Unit, First Affiliated Hospital, Zhengzhou University, Zhengzhou, Henan, PR China; Inserm U837, FRANCE

## Abstract

Curcumin, the most active component of turmeric, has various beneficial properties, such as antioxidant, anti-inflammatory, and antitumor effects. Previous studies have suggested that curcumin reduces the levels of amyloid and oxidized proteins and prevents memory deficits and thus is beneficial to patients with Alzheimer’s disease (AD). However, the molecular mechanisms underlying curcumin’s effect on cognitive functions are not well-understood. In the present study, we examined the working memory and spatial reference memory in rats that received a ventricular injection of amyloid-β1-42 (Aβ1-42), representing a rodent model of Alzheimer’s disease (AD). The rats treated with Aβ1-42 exhibited obvious cognitive deficits in behavioral tasks. Chronic (seven consecutive days, once per day) but not acute (once a day) curcumin treatments (50, 100, and 200 mg/kg) improved the cognitive functions in a dose-dependent manner. In addition, the beneficial effect of curcumin is accompanied by increased BDNF levels and elevated levels of phosphorylated ERK in the hippocampus. Furthermore, the cognition enhancement effect of curcumin could be mimicked by the overexpression of BDNF in the hippocampus and blocked by either bilateral hippocampal injections with lentiviruses that express BDNF shRNA or a microinjection of ERK inhibitor. These findings suggest that chronic curcumin ameliorates AD-related cognitive deficits and that upregulated BDNF-ERK signaling in the hippocampus may underlie the cognitive improvement produced by curcumin.

## Introduction

Alzheimer's disease (AD) is the most common form of dementia, accounting for approximately 50% to 70% of typical, late-onset cases of dementia. AD is histologically characterized by the extracellular deposition of amyloid β peptides (Aβ) and the intracellular accumulation of hyper-phosphorylated tau [1]. Numerous *in vitro* and *in vivo* studies have suggested that Aβ plays a causal role in AD pathogenesis [2, 3]. Notably, Aβ has also been shown to play an important role in the cognition deficits, neuro-inflammation, and apoptosis observed in AD, but the underlying mechanisms remain largely uncertain.

Curcumin, a major polyphenol from curry spice (*Curcumalonga*), has been reported to reverse Aβ detrimental effects in vitro [4, 5]. Previous studies have shown that curcumin possesses many properties that may prevent or ameliorate the pathological processes underlying age-related cognitive decline, dementia or mood disorders. In particular, experimental evidence has demonstrated that curcumin exhibits neuroprotective effects in a variety of AD models [6, 7]. The prevention of synaptic protein loss has been suggested to mediate the protective effects of curcumin [8, 9]. Recently, Hoppe JB and colleagues have shown that Aβ exerted deleterious effects on synaptic activity in hippocampal slice cultures that were counteracted by curcumin via the modulation of synaptic proteins, such as CaMKII and synapsin I [10]. A follow-up study from the same group indicated that rats with Aβ1–42 lateral ventricle infusion showed significant hippocampus-dependent learning and memory impairment (Y-maze task and novel object recognition task), which was associated with a significant decrease in the hippocampal synaptophysin levels, as well as a disturbance in brain-derived neurotrophic factor (BDNF) expression [11].

The neurotrophin BDNF plays a diverse role in regulating neuronal structure and function in both the developing and adult CNS, and it has emerged as one of the most important signaling molecules for the developing nervous system, the impaired nervous system, and multiple diseases, including AD [12, 13]. As a key mediator of synaptic efficacy, BDNF has been shown to be involved in neuronal connectivity and neuroplasticity [14, 15]. Notably, BDNF promotes the survival of basal forebrain cholinergic neurons both *in vitro* and *in vivo* [16, 17]. The selective loss of basal forebrain cholinergic neurons and their projection regions, the hippocampal formation and cortex, are generally thought to be important neuroanatomical markers in AD patients [18, 19]. Several studies have demonstrated altered BDNF serum levels in AD patients. Indeed, BDNF plays a critical role in neuronal survival, synaptic plasticity, and memory [20], all of which are related to AD-related cognitive deficits. Laske C and colleagues have shown that higher BDNF serum levels are associated with a slower rate of cognitive decline in AD patients [21].

Previous studies have suggested that BDNF supports neuronal survival, participates in neuronal plasticity, and mediates long-term potentiation and memory processes, which may contribute to the beneficial effect of curcumin on Aβ-related cognitive dysfunctions. However, direct evidence of this support remains lacking. Therefore, the present study was aimed to investigate whether the BDNF signaling pathway is directly involved in the protective effect of curcumin on cognitive decline in AD and to identify this disease’s underlying mechanisms.

## Material and Methods

### Reagents and drugs

Curcumin and amyloid-β1–42 (Aβ1–42) were obtained from Sigma-Aldrich (St. Louis, MO, USA). Prior to injection, the Aβ1–42 peptide was dissolved in a physiological saline solution at a concentration of 5 mg/ml and incubated at 37°C for 72 h to induce aggregation. Human full-length BDNF proteins and rabbit monoclonal antibodies against phospho-GSK 3β (Ser 9) and total GSK 3β were purchased from Abcam (Cambridge, UK). Rabbit polyclonal antibodies against BDNF, phospho-ERK (Thr202/Tyr204), total ERK, phospho-JNK (Thr183/Tyr185), total JNK, phospho-p38 (Thr180/Tyr182), and total p38 and GAPDH were obtained from Cell Signaling Technology (Beverly, MA, USA). The GSK-3 modulators, wortmannin, and ERK inhibitor PD98059 were obtained from Sigma-Aldrich (St. Louis, MO, USA). We used commercial lentiviral-BDNF shRNA particles and control non-targeting shRNA lentiviral particles, both of which were obtained from Santa Cruz (Santa Cruz Technology, CA, USA).

### Animals

Male Sprague-Dawley rats, weighing 200 ± 20 g at the beginning of the experiment, were housed in a room maintained at 23°C with a 12-hour light-dark cycle. The rats were given free access to food and water. This study was carried out in strict accordance with the recommendations in the Guide for the Care and Use of Laboratory Animals of the National Institutes of Health. The protocol was approved by the Committee on the Ethics of Animal Experiments of the University of Zhengzhou (Permit Number: 2014097B). All surgery was performed under chloral hydrate anesthesia, and all efforts were made to minimize suffering.

### Surgery and microinjection

The animal model of Alzheimer's disease was prepared using the Aβ1–42 aggregates intracerebroventricular (i.c.v.) injection method. The rats were anesthetized with chloral hydrate (35 mg/kg, i.p.) and penicillin (1.5 × 10^5^ U/rat) and mounted on a stereotaxic apparatus (RWD Life Science Co., Ltd, Shenzhen, China). The scalp was incised and retracted, and the head position was adjusted to place bregma and lambda in the same horizontal plane. The skulls of the rats were opened, and burr holes were drilled at the corresponding position to allow for the i.c.v. injection of Aβ1–42 (anteroposterior: -0.8 mm from Bregma, medial/lateral: ±1.4 mm and dorsal/ventral: -4.0 mm) or intra-hippocampal injection (anteroposterior: -3.8 mm from Bregma, medial/lateral: ±2.2 mm and dorsal/ventral: -2.7 mm). For the infusion of Aβ1–42, two small holes were made, and Aβ1–42 (2.0 μl per side) was injected bilaterally into the lateral ventricles through a stainless steel cannula using a Hamilton microsyringe. Sham rats were bilaterally injected with same volume of saline (2.0 μl per side, i.c.v.) into cerebroventricular. The injection lasted 5 min, and the needle with the syringe was left in place for 2 min after the injection to ensure the complete infusion of the drug. After surgery, two stainless steel obturators were inserted into the guides to prevent cannula occlusion. Penicillin was applied daily, and the rats were allowed 5 days to recover from surgery. Behavioral testing was performed 5 days after the surgery (Day 6). Each rat was only used in one behavioral test unless specified.

For the intra-hippocampal injection, two guide cannulas (21-gauge) were inserted into the hippocampus and anchored to the skull with sterile stainless steel screws and acrylic dental cement. BDNF (1.0 μg), PD98059 (20 μM), wortmannin (100 μM) or the same volume of 0.9% saline was infused bilaterally (in a total volume of 1.0 μl/side) into the hippocampus over 5 min. When needed, lentiviral-shBDNF (approximately 1.0 × 10^6^ infectious units of virus per μl) or lentiviral-control shRNA particles were microinjected bilaterally into the hippocampus through the guide cannulas. After completing the injection, the injectors were left in place for 2 min to ensure adequate diffusion from the injector tip. Microinjections were performed 30 min before the behavioral test.

### Y-maze test

To assess working memory and exploration behavior, rats from both the acute and repeated treatment groups were tested in the Y-maze. The Y-maze consisted of three white plastic arms separated by a 120° angle. Each arm was 30 cm long, 10 cm wide, and restricted by 20-cm high walls. The room was moderately lit (200 lux). The animals were placed at the center of the maze and allowed to explore freely for 5 minutes, during which the number and order of arm entries was recorded. According to Typlt *et*. *al* [22], spontaneous changes, which reflect the strength of working memory, were calculated as the ratio between (sequence of 3 consecutive arm entries)/(total arm entries -2). A low percentage of alternation is indicative of an impaired spatial working memory because the rat cannot remember which arm it has just visited, and thus shows decreased spontaneous alternation.

### Open-field test (OFT)

To verify the effects of i.c.v. treatment with Aβ1–42 on locomotor activity, OFT were performed after the Y-maze test. The animals were placed at the center of the arena and allowed to explore the apparatus freely for 60 minutes with the experimenter out of the animal’s sight. A black square arena (100 × 100 × 60 cm) was used for the test. The total distance travelled was analyzed using video-tracking software (SMART, Panlab SL, Barcelona, Spain).

### Morris water maze task

**Fig 1:**
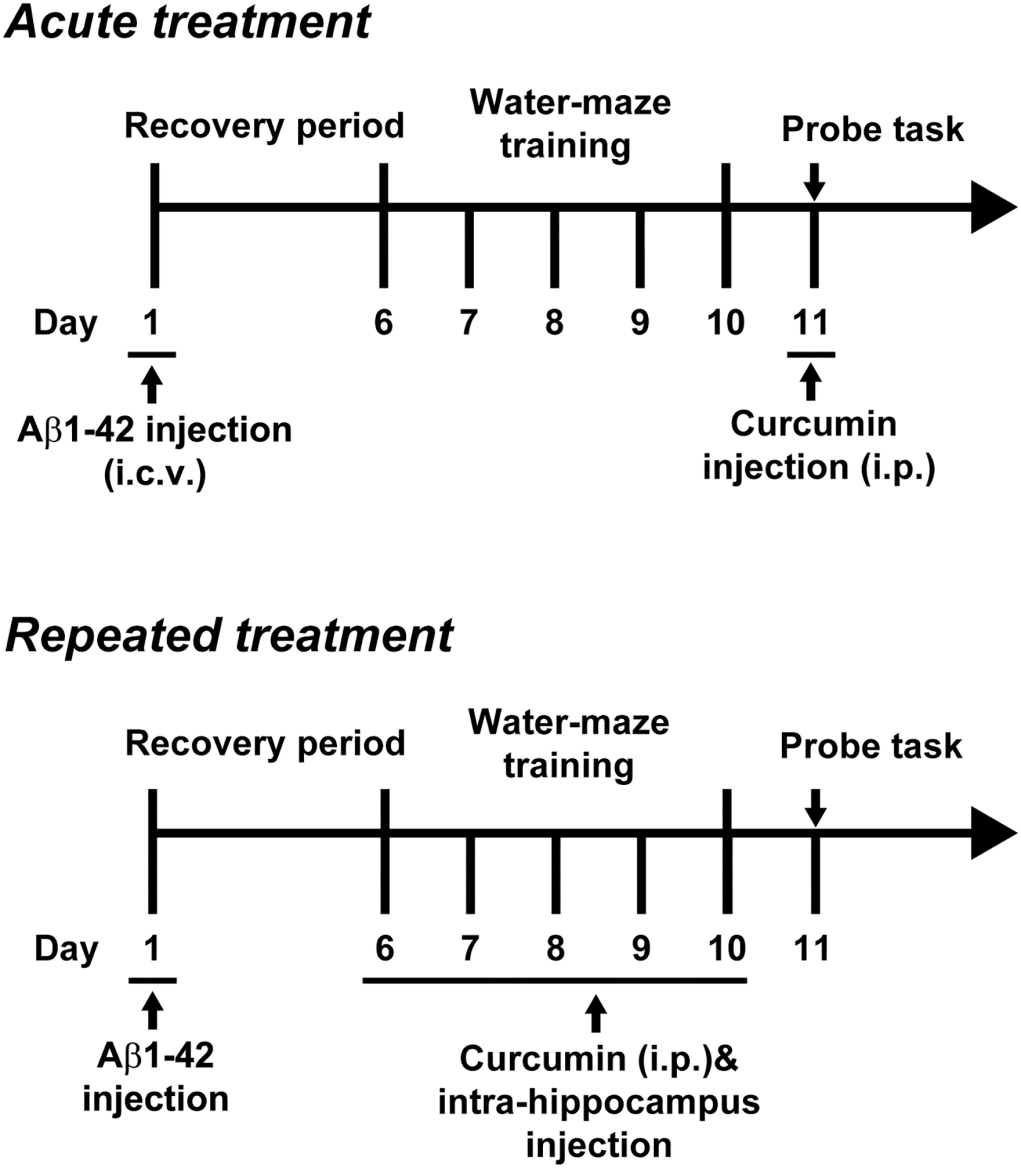
The experimental protocol of the Morris water maze for acute or repeated curcumin treatment.

To assess spatial reference learning and memory, a separate cohort of rats from both the acute and repeated treatment groups were tested in the Morris water maze. The experimental apparatus (RWD Life Science, Shenzhen, China) consisted of a circular water pool (diameter 150 cm; height 60 cm; containing water at 24±2°C) divided into four equally spaced quadrants. The pool was placed in a test room containing various prominent visual cues. A translucent 10 × 10 cm platform submerged 1 cm below the water surface was hidden at the center of quadrant II during the training period and was then removed at the time of the probe task. Memory training was performed 5 days after Aβ1–42 injection. The training was conducted twice per day for five consecutive days before the probe task. Each rat was allowed to swim until it found the platform or until 120 s elapsed. The rat was then left on the platform for 10 s. During the spatial probe task, the platform was removed from the pool, and the rats were allowed to swim for 120 s. The swim escape latency, average swim speed, time spent in the target quadrant, and number of times the animal crossed the previous location of the platform were recorded by a video tracking system (SMART, Panlab SL, Barcelona, Spain). The experimental procedures for acute and chronic curcumin treatment are outlined in Fig 1A.

### Western blot

The rats were sacrificed by decapitation, and the brains were quickly removed. The hippocampal tissues were carefully dissected on ice. To extract the protein, frozen tissues were homogenized in pre-cooled RIPA buffer (50 mM Tris-HCl, 50 mM NaCl, 5 mM EDTA, 10 mM EGTA, 2 mM sodium pyrophosphate, 4 mM paranitrophenylphosphate, 1 mM sodium orthovanadate, 1 mM phenylmethylsulfonyl fluoride, 2 μg/ml aprotinin, 2 μg/ml leupeptin and 2 μg/ml pepstatin, pH 7.5). The homogenates were incubated on ice for 30 minutes and centrifuged at 12000 × *g* and 4°C for 15 min. The protein content was determined using the bicinchoninic acid method (Joincare Co., Zhuhai, China). The protein samples were subjected to 12% SDS-PAGE and transferred to PVDF membranes. The membranes were blocked for 1 h with 5% non-fat milk in Tris-buffered saline (500 mM NaCl, 20 mM Tris-HCl, pH 7.5) containing 0.05% Tween-20 and incubated overnight with primary antibodies at 4°C (all diluted at 1:1000). The next day, the membranes were washed three to four times with 0.1% Tween-20 TBS (pH 7.6) and incubated with horseradish peroxidase-conjugated anti-rabbit or anti-mouse secondary antibodies. An enhanced chemiluminescence kit (Millipore, MA, USA) was used to detect immunoreactive protein bands. The blots were normalized to GAPDH (1:1000).

### Statistical analysis

A statistical analysis was performed using one- or two-way ANOVA followed by Dunnett’s post hoc test, each group was compared with the Aβ1–42+saline group. The results are expressed as the means ± SEM. F, DFn, DFd and *P* indicate the value of the F-test, the degrees of freedom of the numerator and denominator and the significance, respectively, and were used to determine whether the factors significantly affected the result. In the probe tests, one-sample t-tests was performed vs. the 25% hazard level. To analyze the western blots, the detected bands were densitometrically quantified using QuantityOne (Bio-Rad). The relative protein phosphorylation was expressed as the mount of phosphorylated protein *vs*. that of the total protein. Significance was accepted at *P* < 0.05.

## Results

### Acute curcumin treatment does not influence the cognitive function in an Aβ1-42-treated rat model of Alzheimer's disease

**Fig 2:**
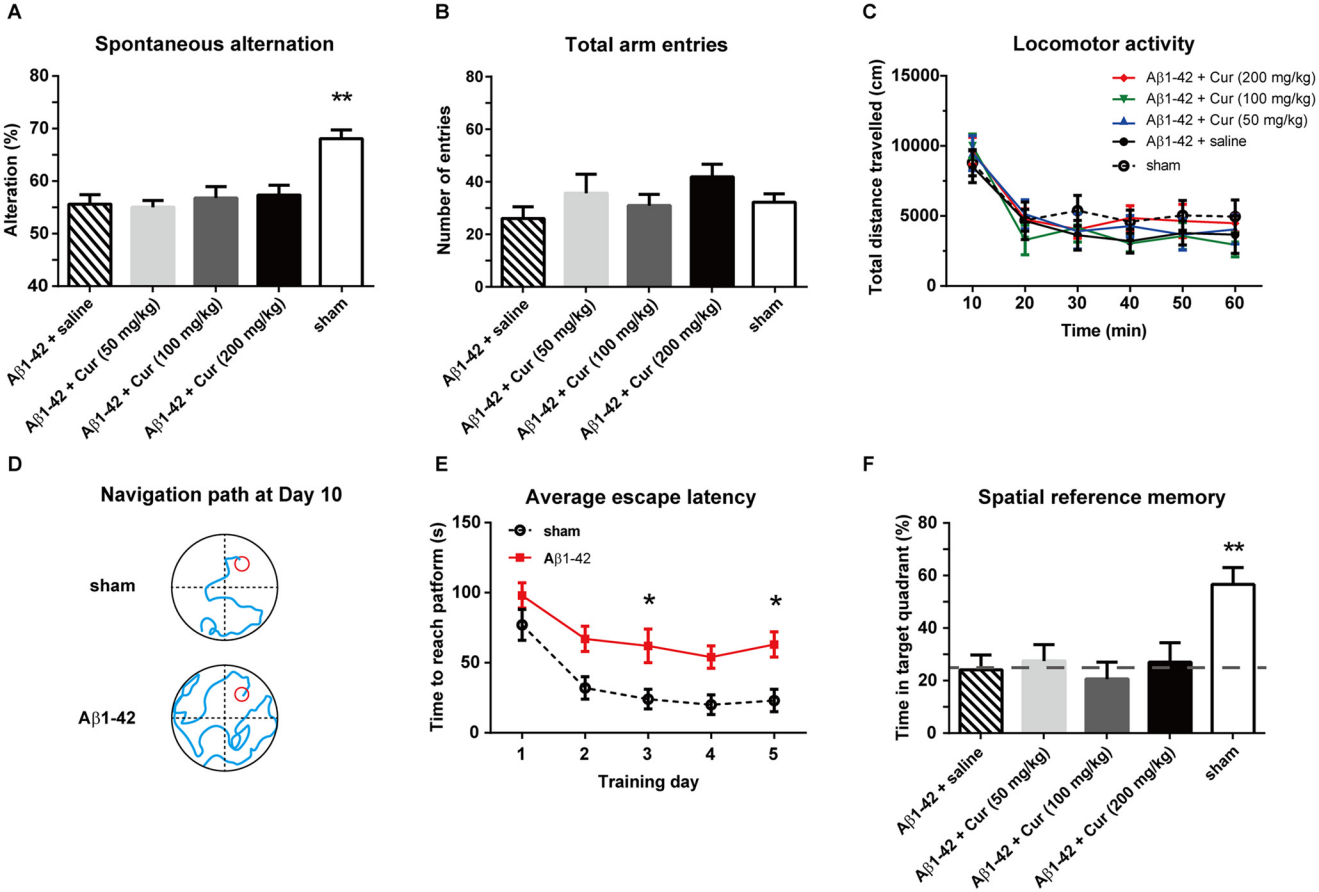
Effect of acute curcumin on working memory, motor function and spatial reference memory in an Aβ1-42-treated rat model of Alzheimer's disease. (A) The spontaneous alteration in the Y-maze. (B) The total arm entries in the Y-maze. (C) The total distance travelled in an open-field test. (D) Representative swim traces in the Morris water maze training trial. (E) The escape latency during the water maze training trials. (F) The time spent in the target quadrant in the probe task. n = 8 each group. * *P* < 0.05 and ** *P* < 0.01 compared with Aβ1–42 + saline group

The rats were injected with a single dose of curcumin (50, 100 and 200 mg/kg, i.p.) or saline (n = 8/group) and underwent behavioral tests. Each rat only participated in one behavioral test. A one-way ANOVA was used to analyze the significance of the effect of treatment on spontaneous alternation [F (4, 35) = 4.981, *P* < 0.01; Fig 2A] but not total arm entries [F (4, 35) = 1.685, *P* = 0.1754; Fig 2B] in the Y-maze. As expected, Aβ1–42+saline rats showed a significant lower ratio of spontaneous alternation (*P* < 0.01) compared with sham (i.c.v. injected with saline) rats, suggesting impaired working memory in the Aβ1-42-induced rat model of Alzheimer's disease. However, Aβ1-42-treated rats injected with a single dose of curcumin of 50, 100 or 200 mg/kg did not show any improvement in spontaneous alternation (*P* = 0.99, 0.97 and 0.89, respectively) compared with Aβ1–42+saline rats. Next, we evaluated whether Aβ1–42 or acute curcumin could affect motor function because almost all cognitive tests rely on motor behavior. Two-way ANOVA with repeated measurements reported a significant effect of time [F (5, 210) = 22.16, *P* < 0.0001] but not treatment [F (4, 210) = 1.403, *P* = 0.234] on the total distance in OFT (Fig 2C), indicating that these rats do not exhibit locomotor deficits. We then tested spatial reference learning and memory in a Morris water maze. The representative navigation paths at the end of the water-maze training (day 10, ie. the training day 5) demonstrated that spatial learning acquisition was obviously impaired in the Aβ1-42-treated rats relative to the rats of the sham control group (Fig 2D). Further analysis revealed that the escape latencies decreased from training day 1 to day 5 in both the sham and Aβ1-42-treated groups [F_time_ (4, 190) = 4.307, *P* < 0.01] (Fig 2E). Moreover, the i.c.v. administration of Aβ1–42 significantly attenuated the spatial learning ability of rats [F_treatment_ (1, 190) = 14.70, *P* < 0.01]. The Aβ1-42-treated rats showed longer escape latencies than the sham rats on training days 3 and 5 (both *P* < 0.05). Twelve hours after the last water maze training, a single dose of curcumin was administered i.p., and the rats were subjected to the probe task to assess the spatial reference memory (Fig 2F). One-way ANOVA revealed a main effect of treatment on the time spent in the target quadrant [F (4, 35) = 5.022, *P* < 0.01]. However, the Aβ1–42+saline group did not differ from the Aβ1–42 combined with 50, 100 or 200 mg/kg curcumin groups (*P* = 0.98, 0.97 and 0.99, respectively), indicating that acute curcumin administration did not improve spatial reference memory. Moreover, sham group was significantly increased from the Aβ1–42+saline group (*P* < 0.01) and 25% hazard level (*P* < 0.0001). In addition, the visible platform version of MWM was performed to detect visual and motivational deficits. The results indicated no visual and motivational deficits in Aβ1–42 rats (S1 Fig).

### Chronic curcumin treatment improves cognitive function and promotes the expression of BDNF and phospho-ERK in hippocampus

**Fig 3:**
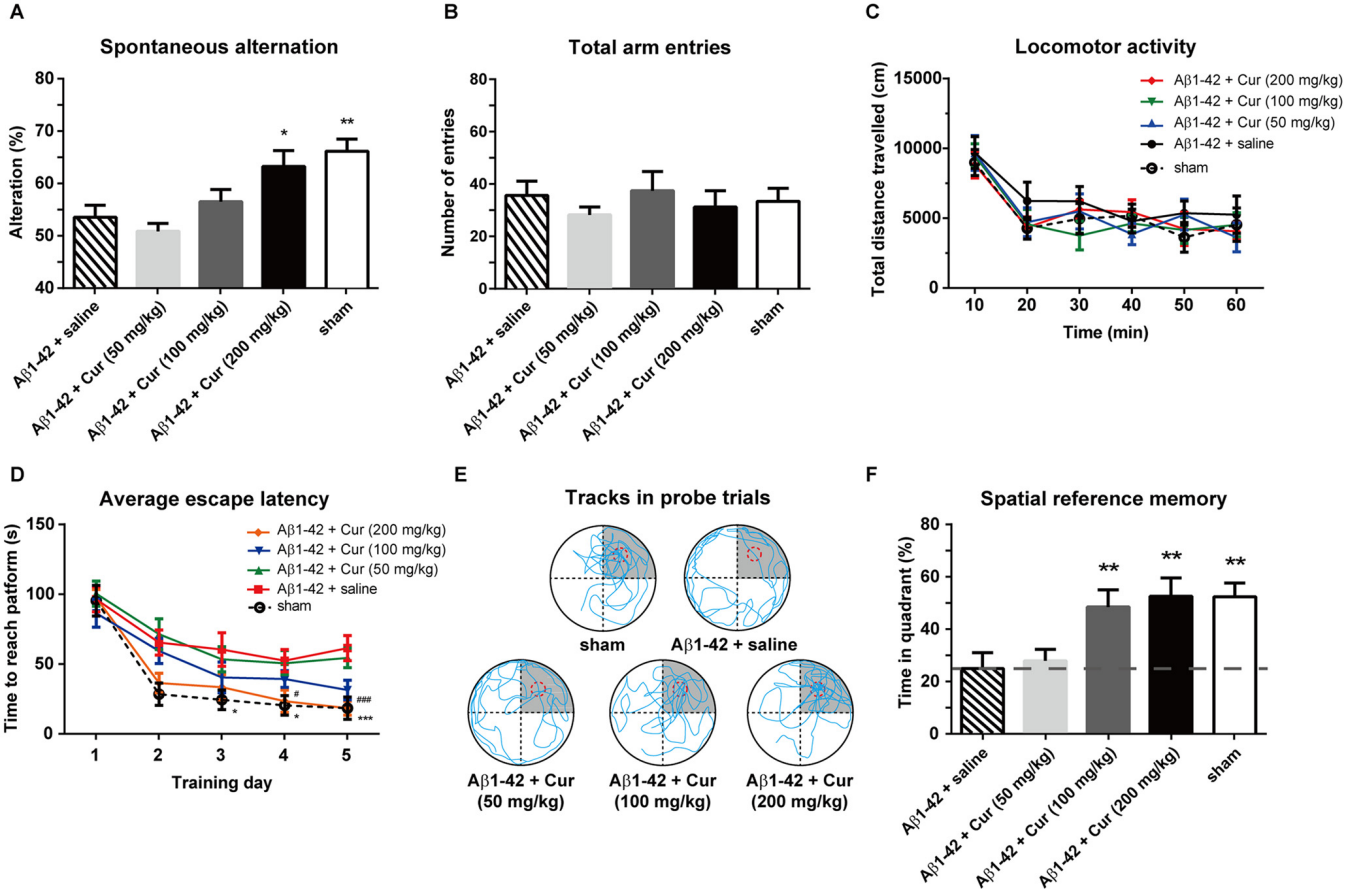
Effect of chronic curcumin treatment on working memory, motor function and spatial reference memory in Alzheimer's disease rats. (A) The spontaneous alteration in the Y-maze. (B) The total arm entries in Y-maze. (C) The total distance travelled in open-field test. (D) The escape latency during the water maze training trials. (E) Representative swim traces in the probe task. For panel D, *P < 0.05 and ***P < 0.0001 comparison between Aβ1–42 + saline and sham; # *P* < 0.05 and ###*P* < 0.0001 comparison between curcumin (200 mg/kg) and Aβ1–42 + saline. (F) The time spent in the target quadrant in the probe task. n = 8 each group. **P < 0.01 and ***P < 0.0001 compared with Aβ1–42 + saline.

Rats were treated with curcumin (50, 100 and 200 mg/kg, i.p., once per day) or saline (n = 8/group) for 5 consecutive days. Each rat only participated in one of the behavioral tests, including the Y-maze, OFT or Morris water maze. Chronic treatments significantly affected the spontaneous alternation [F (4, 35) = 6.046, *P* < 0.01; Fig 3A] but not total arm entries [F (4, 35) = 0.5965, *P* = 0.6675; Fig 3B]. The spontaneous alternation in the Aβ1–42+Cur (50 mg/kg) and Aβ1–42+Cur (100 mg/kg) groups were not differ from the Aβ1–42+saline group (*P* = 0.98 and 0.95, respectively). However, there was a significant increase of the spontaneous alternation in the 200 mg/kg curcumin-treated rats (*P* < 0.05), indicating an amelioration in working memory. A significant effect of time [F (5, 210) = 18.08, *P* < 0.0001] but not treatment [F (4, 210) = 1.125, *P* = 0.3459] on the total distance of OFT was observed, suggesting no locomotor deficits in these rats (Fig 3C). The escape latencies significantly decreased after the chronic administration of 100 and 200 mg/kg curcumin [F_treatment_ (4, 295) = 4.813, *P* < 0.01; F_time_ (4, 295) = 15.97, *P* < 0.0001; Fig 3D]. The representative navigation paths in the probe trials are shown in Fig 3E. In the probe trials, chronic curcumin treatment significantly affected the time spend in the target quadrant [F (4, 35) = 4.342, *P* < 0.01; Fig 3F]. The time spent in the target quadrant in the sham and Aβ1–42+Cur (100 and 200 mg/kg)-treated rats were significantly higher than the Aβ1–42+saline rats (all *P* < 0.01), indicating that the spatial memory markedly improved in the Alzheimer's disease rats. Moreover, Aβ1–42+Cur (100 and 200 mg/kg) and the sham group were significantly higher vs the 25% hazard level (*P* < 0.0001 of all groups). To rule out the possible effect of curcumin per se on the spatial memory, we conducted the chronic curcumin administration without Aβ injection (ie. sham+Cur group) to address this concern. No significant effect of curcumin (50, 100 and 200 mg/kg) was found on the spatial learn memory (S2 Fig), indicating that the chronic curcumin administration per se do not affect spatial memory.

**Fig 4:**
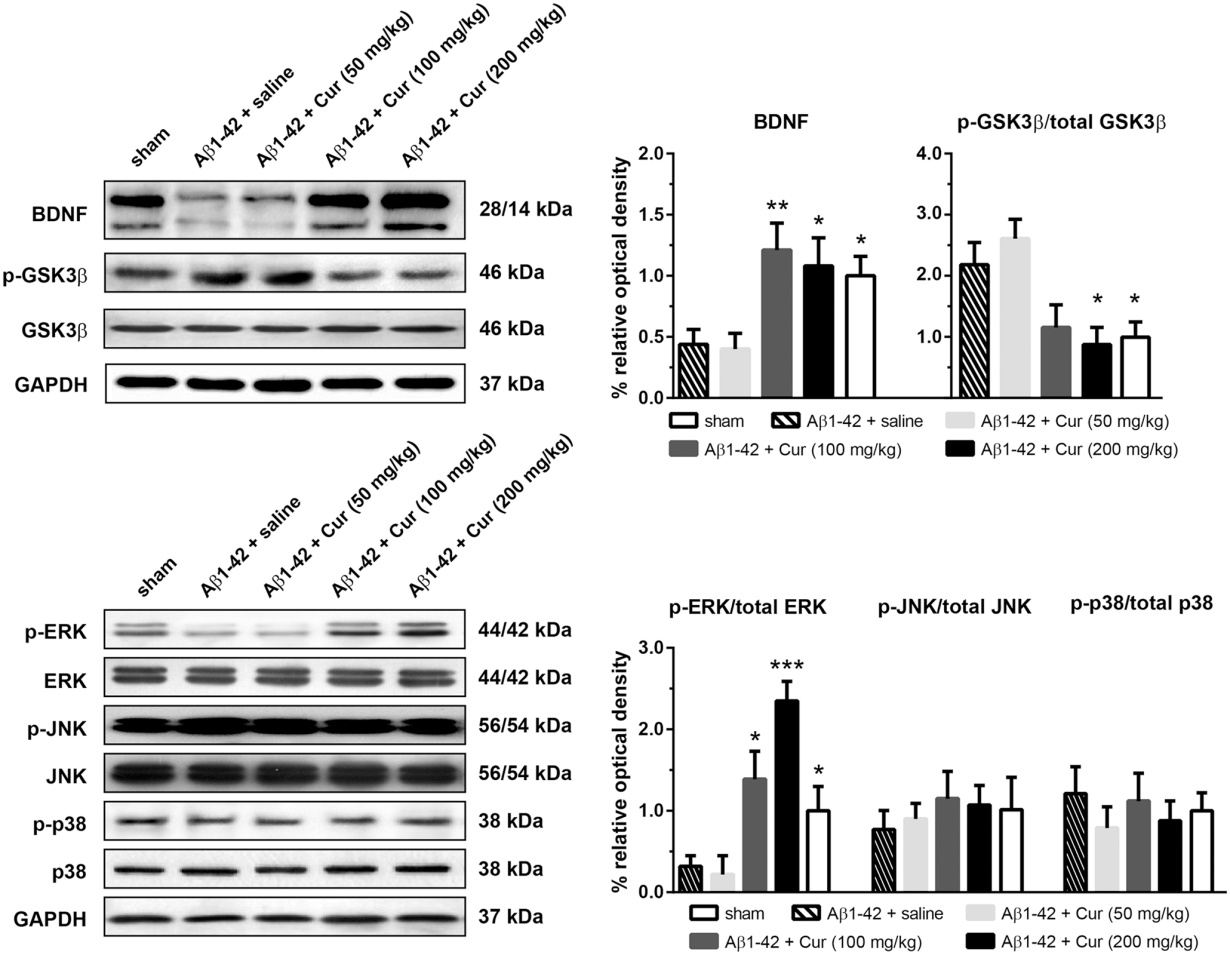
Effect of chronic curcumin treatment on BDNF expression and GSK3β, MAPK phosphorylation in the hippocampus. Upper-left: representative western blots of BDNF and GSK3β in each group. GAPDH was used as the internal loading control. Upper-right: relative expression of BDNF expression phosphorylated GSK3β. Lower-left: representative western blots of ERK, JNK and p38 phosphorylation in each group. Lower-right: relative expression of phosphorylated ERK, JNK and p38. Protein phosphorylation was expressed as phosphorylated form vs. total protein. * *P* < 0.05 and ** *P* < 0.01 compared with Aβ1–42 + saline.

To observe the effects of chronic curcumin on BDNF, GSK3β and MAPK activation in the Aβ1-42-treated rat model of Alzheimer's disease, a new cohort of animals was injected with curcumin (i.p., once per day) for 7 consecutive days and killed 30 min after the last injection. The expression of levels BDNF, total GSK3β, ERK, JNK and p38, as well as their phosphorylated (activated) forms in the hippocampus were analyzed (Fig 4). Treatment significantly affected the BDNF expression in the hippocampus [F (4, 35) = 4.525, *P* = 0.0047]. The expression of BDNF was significantly promoted in the Aβ1–42+Cur (100 and 200 mg/kg, *P* < 0.01 and 0.05) and the sham group (*P* < 0.05) as compared with the Aβ1–42+saline group. However, the repeated administration of 50 mg/kg curcumin did not affect the BDNF levels (*P* = 0.987). The phosphorylation of GSK3β [F (4, 35) = 5.328, *P* = 0.0019] was significantly decreased by repeated administration of 100 and 200 mg/kg curcumin compared with the Aβ1–42+saline groups. Furthermore, there was no difference in phospho-GSK3β between the Aβ1–42+saline and the Aβ1–42+Cur (50 mg/kg) group (*P* = 0.8478). The level of total GSK3β did not differ among groups [F (4, 35) = 0.5644, *P* = 0.7509]. A one-way ANOVA revealed a significant effect of treatments on the phosphorylation of ERK [F (4, 35) = 11.37, *P* < 0.0001] but not on JNK [F (4, 35) = 0.2659, *P* = 0.8978] or p38 [F (4, 35) = 0.3674, *P* = 0.8302]. A post hoc analysis showed that the levels of phospho-ERK in the Aβ1–42+Cur (100 and 200 mg/kg, *P* < 0.05 and 0.0001) and the sham group (*P* < 0.05) were significantly higher than in the Aβ1–42+saline group. Phospho-ERK in the Aβ1–42+Cur (50 mg/kg) was similar to the sham group (*P* = 0.9691).

### BDNF in the hippocampus is necessary for the cognition enhancement effect of chronic curcumin

**Fig 5:**
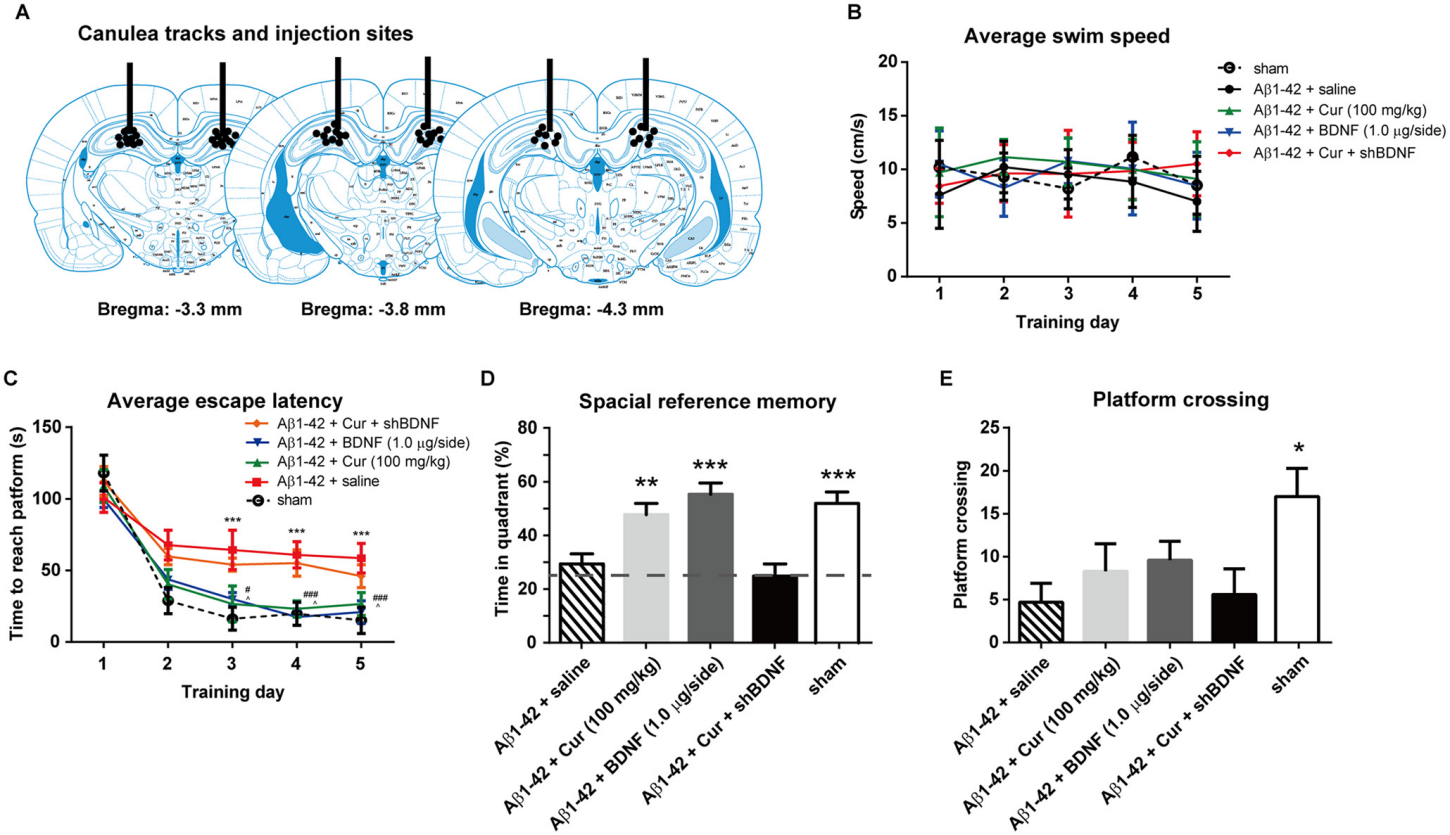
Effect of intra-hippocampal injections of BDNF protein or lentiviral-shBDNF on spatial learning function in the rat model of Alzheimer's disease. (A) Schematic representation showing the approximate location of microinjections into the VLO. Only rats with injection cannula tips located bilaterally in this site were included. (B) Swim speed in each training trial. (C) The escape latency during the water maze training trials. (D) The time spent in the target quadrant and (E) the number of times crossing the platform in the probe task. n = 8/group. For panel C, ***P < 0.0001, Aβ1–42 + saline *vs* sham; # *P* < 0.05, ### *P* < 0.0001, Aβ1–42 + BDNF *vs*. Aβ1–42 + saline group; ^ *P* < 0.05, Aβ1–42 + Cur *vs*. Aβ1–42 + saline group. For other panels, * *P* < 0.05, ** *P* < 0.01, *** *P* < 0.0001 compared with Aβ1–42 + saline.

Chronic curcumin (100 mg/kg, i.p.) or saline (sham rats) were administered as previously described. BDNF (1.0 μg/side) or lentiviral-shBDNF was injected bilaterally into the hippocampus 30 min after curcumin injection. At the end of the behavioral test, the rats were killed, and their brains were removed. The cannula tracks and injection sites in the hippocampus were verified, and an example is shown in Fig 5A. The cannula lesion and injection sites were well localized to the dorsal hippocampus. Thirty-eight animals in total were included in the final analyses (sham group, n = 7; Aβ1–42+saline group, n = 8; Aβ1–42+Cur group, n = 8; Aβ1–42+BDNF group, n = 7; Aβ1–42+Cur+shBDNF group, n = 8). Two rats were discarded due to misplaced injection sites. No changes in swimming speed were observed in these rats during the entire water maze training period [F_treatment_ (4, 167) = 0.1839, *P* = 0.9465; F_time_ (4, 167) = 0.1581, *P* = 0.9591] (Fig 5B), indicating no locomotor deficits in these rats. Two-way ANOVA revealed a significant effect of drug treatments [F_treatment_ (4, 283) = 4.246, *P* = 0.0023] as well as time [F_time_ (4, 283) = 20.64, *P* < 0.0001] on the escape latency (Fig 5C). In the probe trial, the amount of time in the quadrant was significantly higher in the Aβ1–42+Cur, Aβ1–42+BDNF and the sham group than in the Aβ1–42+saline group (*P* < 0.01, 0.0001 and 0.0001, respectively) (Fig 5D). Moreover, Aβ1–42+Cur, Aβ1–42+BDNF and the sham group were significantly higher vs the 25% hazard level (*P* < 0.0001 of all groups). No obvious difference in the platform crossing was observed between the Aβ1–42+saline, Aβ1–42+Cur, Aβ1–42+BDNF and Aβ1–42+Cur+shBDNF groups (Fig 5E). Moreover, the amount of time in the quadrant and number of times the platform crossed did not significantly differ between the Aβ1–42+salineand Aβ1–42+Cur+shBDNF rats, indicating that intra-hippocampus shBDNF administration completely blocked chronic curcumin-induced cognitive improvement.

### Intra-hippocampus infusion of ERK inhibitors, but not GSK3 activator, blocks the curcumin-induced cognitive improvement

**Fig 6:**
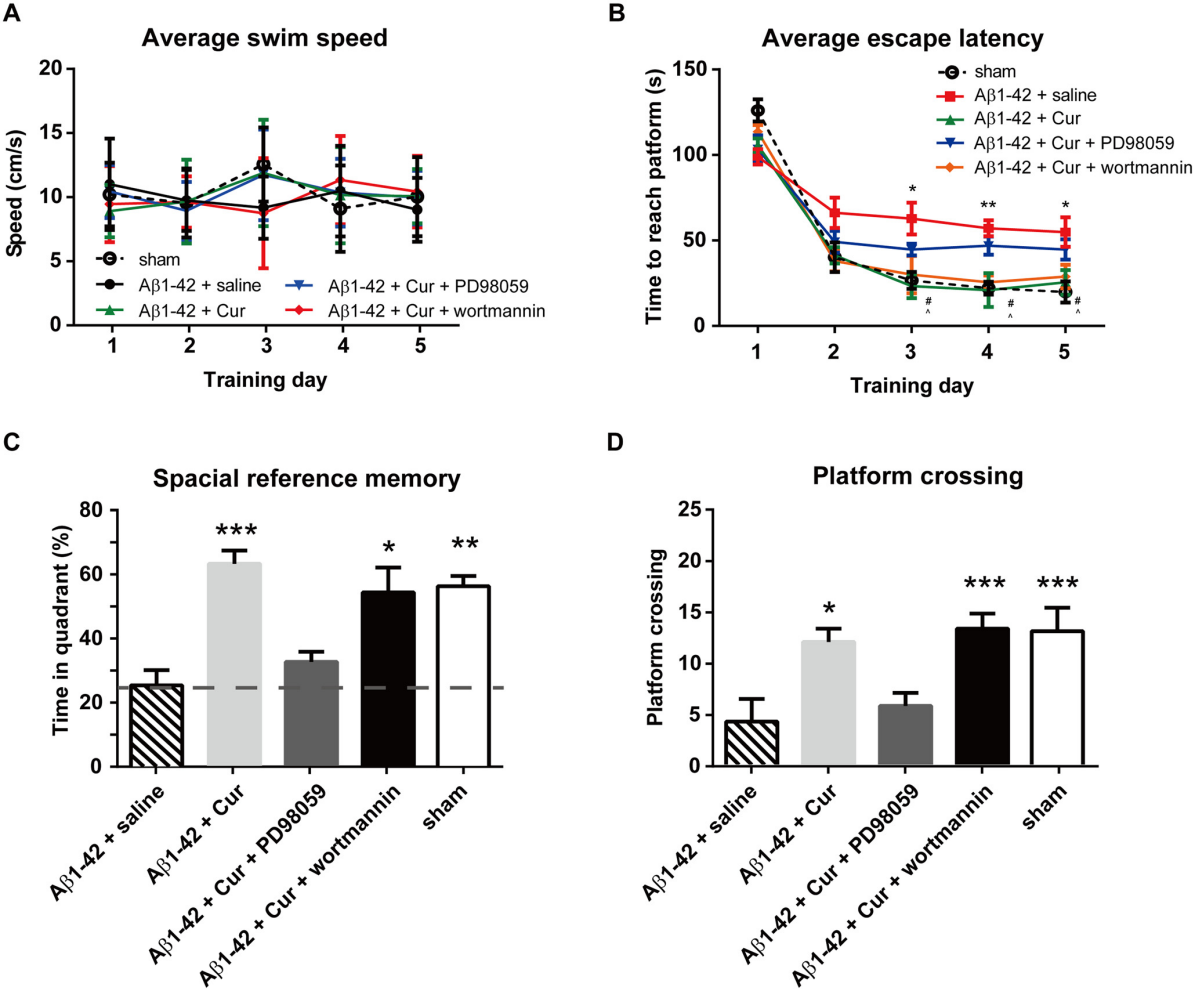
Effect of intra-hippocampal injections of ERK inhibitor or GSK3 activator on spatial learning function in the rat model of Alzheimer's disease. The ERK inhibitor PD98059 (20 μM) or GSK3 activator wortmannin (100 μM) (combined with chronic curcumin i.p. injection) was injected bilaterally into the hippocampus 30 min before the water-maze training trial. (A) Swim speed in each training trial. (B) The escape latency during the water maze training trials. (C) The time spent in the target quadrant and (D) the number of times crossing the platform in the probe task. n = 8/group. For panel B, *P < 0.05, **P < 0.01, Aβ1–42 + saline *vs*. sham control group; # *P* < 0.05, Aβ1–42 + Cur *vs*. Aβ1–42 + saline group; ^ *P* < 0.05, Aβ1–42 + Cur + wortmannin *vs*. Aβ1–42 + saline group. For other panels, * *P* < 0.05, ** *P* < 0.01, *** *P* < 0.0001 compared with Aβ1–42 + saline.

Because chronic curcumin affected both hippocampal ERK and GSK3β phosphorylation, we next investigated which of these factors plays a larger role in curcumin-induced hippocampus-dependent spatial memory enhancement. The ERK inhibitor PD98059 (20 μM) or GSK3 activator wortmannin (100 μM) (combined with chronic curcumin i.p. injection) was injected bilaterally into the hippocampus 30 min before the water-maze training trial. Thirty-six animals total were included in the final analyses (sham group, n = 8; Aβ1–42+saline group, n = 6; Aβ1–42+Cur group, n = 8; Aβ1–42+Cur+PD98059 group, n = 6; Aβ1–42+Cur+wortmannin group, n = 8). Four rats were discarded due to misplaced injection sites. No changes in swimming speed were observed in these rats during the entire water maze training period [F_treatment_ (4, 159) = 0.02157, *P* = 0.9991; F_time_ (4, 159) = 0.1366, *P* = 0.9686] (Fig 6A). A two-way ANOVA revealed a significant effect of drug treatments on [F_treatment_ (4, 159) = 10.95, *P* < 0.0001], time [F_time_ (4, 159) = 110.7, *P* < 0.0001] and an interaction [F (16, 159) = 2.583, *P* = 0.0013] with the escape latency (Fig 6B). In the probe trial, the rats in the Aβ1–42+Cur, Aβ1–42+Cur+wortmannin and sham groups spent increased amounts of time in the quadrant (Fig 6C) and crossed the platform at similar frequencies (Fig 5D) compared with the Aβ1–42+saline group. However, the Aβ1–42+Cur+PD98059 group showed no difference in both the time spent in the quadrant and platform crossing frequency compared with the Aβ1–42+saline rats. The Aβ1–42+Cur (*P* < 0.0001), Aβ1–42+Cur+wort (*P* < 0.01) and the sham group (*P* < 0.01) were significantly higher vs the 25% hazard level. These results suggest that an intra-hippocampus infusion of ERK inhibitors, but not GSK3 activator, blocks the curcumin-induced cognitive improvement.

## Discussion

In this study, we found that an intracerebroventricular injection of Aβ1–42 decreased the BDNF levels in the hippocampus of rats that exhibited impaired performances in the Y-maze and water maze. Repeated curcumin administration exerted an obvious protective effect on the cognitive functions of Aβ-treated rats, and the effects were associated with increased BDNF and pERK levels in the hippocampus. Furthermore, the overexpression of BDNF in the hippocampus of Aβ-injected rats mimics the effect of curcumin on cognitive impairments, which can be blocked by ERK inhibitor. Therefore, curcumin benefits AD-related cognitive functions mainly via BDNF-ERK signaling activation.

Cognitive decline in patients with AD is associated with elevated brain levels of Aβ, particularly neurotoxic Aβ1–42. The direct Aβ1–42 administration into the brains of rodents has been shown to cause obvious memory deficits that mimic the cognitive decline in AD [23–25]. Previous studies have shown that Aβ1–42 aggregates injected icv can be detected in the hippocampus for weeks [26]. Nakamura and colleagues have shown that icv injected Aβ1–42 can induce progressive learning impairments even 80 days after the treatment which associated with decreased hemicholinium-3 (HC-3) binding and choline acetyltransferase (ChAT) hypoactivity [25]. In addition, using immunohistochemistry, we have detected Aβ1-42 in the hippocampus 7 days after injection (S3 Fig). The cognitive deficits can be assessed with several behavioral tasks, such as spontaneous alternations in the Y-maze and novel object recognition tasks [11]. Moreover, previous studies [26, 27] have shown that Aβ-exposed rats exhibit obvious hippocampus-dependent spatial learning and memory deficits both in short-term and long-term tasks. The Morris water maze is one of the most widely used tests to measure hippocampal-dependent spatial-based learning and memory. Our results demonstrated that Aβ treatment caused significant learning and memory deficits in the water maze performance, while curcumin treatment could dramatically reverse these cognitive alterations. In addition, the locomotor activity in the open field test did not differ between any of the groups. Thus, the observed impairment of memory of the rats was not attributed to the differences in their locomotion activities.

BDNF deficits have been demonstrated in postmortem studies of AD patients and experimental AD animal models. Multiple groups have observed a decrease in the levels of BDNF protein in the hippocampal formation using either ELISA, immunohistochemistry, or western blot analyses [28–33]. The levels of serum BDNF, which is believed to reflect the levels of BDNF in the brain, decreases in AD patients when compared to age-matched healthy controls [34], and higher levels of serum BDNF are reportedly associated with a slower rate of cognitive decline in AD patients [21]. Consistent with previous results published by Hoppe JB *et al*. [11], our data showed that Aβ-infused rats exhibited impairments in hippocampus-dependent memory. Moreover, our unpublished results also indicated that an intra-hippocampus infusion of BDNF appeared to normalize the change caused by Aβ. Interestingly, the direct infusion of BDNF did not ameliorate age-related learning and memory decline [35, 36]; in fact, transgenic mice that overexpress BDNF exhibit learning deficits [37]. Based on these findings, the BDNF induced by curcumin could directly target the deficits caused by Aβ rather than its effect on general synaptic strength [38].

Several lines of evidence have implicated endogenous BDNF-TrkB signaling in the gene regulatory events that contribute to synaptic plasticity and hippocampus-dependent learning and memory in an ERK-dependent manner. Although the roles of ERK in synaptic plasticity and memory are well-documented [39], the contribution of ERK activation in the etiology of AD is far from clear. Our data have suggested that decreased ERK activation in the hippocampus is associated with the Aβ-related cognitive impairments. ERK shows stage-dependent abnormalities in mRNA and protein expression in AD patients [40] and AD models [41]. Although transient ERK activation plays important roles in memory-related processes, persistent activation can mediate NMDA-related excitotoxicity [42]. Therefore, either the hyper- or hypo-activation of ERK could contribute to disease pathways. Specifically, ERK is extensively activated in the astroglial cells of the white matter in early AD, while this activation is reduced and inversely correlates with the Braak stage as well as the Blessed score for cognition in advanced AD [40]. However, the function of ERK activation was cell type- and brain region-specific. For example, active ERK is a tau kinase that is elevated during the initial stages of neurofibrillary degeneration in the projecting neurons in the transentorhinal region. Nevertheless, the causal role of ERK in neurofibrillary tangle formation remains unclear, as many neurons that show the strongest ERK immunoreactivity do not appear vulnerable to neurofibrillary tangles [43]. Notably, ERK activation is suppressed during the late stages relative to the early stage and normal controls in neuronal cell bodies and dystrophic neurites [40]. These results suggested that stage-dependent ERK activation is followed by loss of active ERK. Notably, although phosphatidylinositol 3-kinase (PI3K) have also been implicated in BDNF signaling, their activation is not required for spatial memory recovery in our AD model rats because the PI3K inhibitor wortmannin could not block the beneficial effect of hippocampal BDNF on long term memory restore in the Aβ1-42-injected rats (data not shown).

Our results indicate that repeated curcumin treatments activated ERK signaling, and these findings are consistent with previous studies [44, 45]. Tong L and colleagues have shown that sub-lethal concentrations of Aβ down-regulate the BDNF-induced activation of critical transcription factors, such as cAMP response element binding protein (CREB), by suppressing the Ras-MAPK/ERK and PI3-K/Akt pathways in cultured cortical neurons [46]. Curcumin could restore the learning and memory ability impaired by Aβ in an AD model by activating the BDNF-ERK-CREB signaling in the hippocampus. Recent studies have shown that Akt/GSK3β signaling is also involved in the beneficial effect of curcumin on Aβ-induced cognitive impairments [11]. GSK3 is a serine–threonine kinase that mediates multiple intracellular signaling pathways in the CNS. Notably, GSK3 plays a negative feedback regulatory role in encoding memory and cognitive processing [47]. GSK-3 interacts with multiple components of the plaque-producing amyloid system associated with Alzheimer's disease (for review see [48]). AKT is the most studied of the protein kinases that can phosphorylate and inactivate GSK3 [49]. Aβ exposure could increase the GSK3-mediated inhibition of CREB phosphorylation and subsequently decrease BDNF expression [50]. In the present study, we observed that an intra-hippocampal infusion of ERK inhibitors, but not GSK3 activator, could block the curcumin-induced cognitive improvement in Aβ-treated rats. These results indicated that ERK, but not GSK3, plays a critical role in hippocampus-dependent spatial memory enhancement in our experimental context. However, Akt/GSK3 signaling may also be involved in the neuroprotective effect of curcumin in other AD models.

In summary, our data suggest that curcumin blocks the Aβ-induced memory deficits in a dose-dependent manner. The overexpression of BDNF in the hippocampus mimics the beneficial effect of curcumin on cognitive decline in the AD model. ERK, but not the GSK signaling, plays an important role in the protective effect of curcumin against Aβ-induced memory impairments. Our study may help to elucidate the mechanism of the protective effect of curcumin against Aβ-induced cognitive dysfunctions and demonstrates that curcumin could be a potential candidate in anti-AD therapy.

## Supporting Information

S1 Fig Visible platform version of MWM of Aβ1–42 injected and sham rats. Performance in the Morris water maze using cued platform training was assessed by (A) escape latency, (B) swim velocity during 5 training days. Each point represents the mean ± SEM for the two trials of each group on each day. n = 6/group.
(TIF)

S2 Fig Effect of chronic curcumin treatment on spatial reference memory in sham rats. Curcumin (50, 100 and 200 mg/kg) was administrated i.p. for 5 consecutive days (Day 6–10) in rats without Aβ injection (ie. the sham+Cur group). (A) Swim speed in each training trial. (B) The escape latency during the water maze training trials. (C) The time spent in the target quadrant and (D) the number of times crossing the platform in the probe task. n = 6/group.
(TIF)

S3 Fig Immunohistochemistry in Aβ1-42-induced model and control. Representative figures of Aβ1-42 immunohistochemistry from model (A) and control groups (B), respectively. Scale bar 20 μm. The black arrows are showing the positive cells from immunostaining.
(TIF)

## References

[1] Acosta D, Wortmann M. Alzheimer’s Disease International World Alzheimer Report 2009. Prince, M. 2009:1–92.

[2] Selkoe DJ. The molecular pathology of Alzheimer's disease. Neuron. 1991;6(4):487–98. Epub 1991/04/01. 0896-6273(91)90052-2 [pii]. . 1673054 10.1016/0896-6273(91)90052-2

[3] Hardy J, Allsop D. Amyloid deposition as the central event in the aetiology of Alzheimer's disease. Trends Pharmacol Sci. 1991;12(10):383–8. Epub 1991/10/01. . 1763432 10.1016/0165-6147(91)90609-v

[4] Qin XY, Cheng Y, Yu LC. Potential protection of curcumin against intracellular amyloid beta-induced toxicity in cultured rat prefrontal cortical neurons. Neuroscience letters. 2010;480(1):21–4. 10.1016/j.neulet.2010.05.062 . 20638958

[5] Ye J, Zhang Y. Curcumin protects against intracellular amyloid toxicity in rat primary neurons. International journal of clinical and experimental medicine. 2012;5(1):44–9. 22328947 PMC3272685

[6] Lim GP, Chu T, Yang F, Beech W, Frautschy SA, Cole GM. The curry spice curcumin reduces oxidative damage and amyloid pathology in an Alzheimer transgenic mouse. The Journal of neuroscience: the official journal of the Society for Neuroscience. 2001;21(21):8370–7. . 11606625 10.1523/JNEUROSCI.21-21-08370.2001 PMC6762797

[7] Begum AN, Jones MR, Lim GP, Morihara T, Kim P, Heath DD, et al Curcumin structure-function, bioavailability, and efficacy in models of neuroinflammation and Alzheimer's disease. The Journal of pharmacology and experimental therapeutics. 2008;326(1):196–208. 10.1124/jpet.108.137455 18417733 PMC2527621

[8] Sun CY, Qi SS, Zhou P, Cui HR, Chen SX, Dai KY, et al Neurobiological and pharmacological validity of curcumin in ameliorating memory performance of senescence-accelerated mice. Pharmacology, biochemistry, and behavior. 2013;105:76–82. 10.1016/j.pbb.2013.02.002 . 23402943

[9] Xu Y, Lin D, Li S, Li G, Shyamala SG, Barish PA, et al Curcumin reverses impaired cognition and neuronal plasticity induced by chronic stress. Neuropharmacology. 2009;57(4):463–71. 10.1016/j.neuropharm.2009.06.010 . 19540859

[10] Hoppe JB, Haag M, Whalley BJ, Salbego CG, Cimarosti H. Curcumin protects organotypic hippocampal slice cultures from Abeta1-42-induced synaptic toxicity. Toxicology in vitro: an international journal published in association with BIBRA. 2013;27(8):2325–30. 10.1016/j.tiv.2013.10.002 . 24134851

[11] Hoppe JB, Coradini K, Frozza RL, Oliveira CM, Meneghetti AB, Bernardi A, et al Free and nanoencapsulated curcumin suppress beta-amyloid-induced cognitive impairments in rats: involvement of BDNF and Akt/GSK-3beta signaling pathway. Neurobiology of learning and memory. 2013;106:134–44. 10.1016/j.nlm.2013.08.001 . 23954730

[12] Barde YA, Davies AM, Johnson JE, Lindsay RM, Thoenen H. Brain derived neurotrophic factor. Progress in brain research. 1987;71:185–9. . 3588941 10.1016/s0079-6123(08)61823-3

[13] Leibrock J, Lottspeich F, Hohn A, Hofer M, Hengerer B, Masiakowski P, et al Molecular cloning and expression of brain-derived neurotrophic factor. Nature. 1989;341(6238):149–52. 10.1038/341149a0 . 2779653

[14] Duman RS, Malberg J, Nakagawa S, D'Sa C. Neuronal plasticity and survival in mood disorders. Biological psychiatry. 2000;48(8):732–9. . 11063970 10.1016/s0006-3223(00)00935-5

[15] Cotman CW, Berchtold NC. Exercise: a behavioral intervention to enhance brain health and plasticity. Trends in neurosciences. 2002;25(6):295–301. . 12086747 10.1016/s0166-2236(02)02143-4

[16] Connor B, Dragunow M. The role of neuronal growth factors in neurodegenerative disorders of the human brain. Brain research Brain research reviews. 1998;27(1):1–39. . 9639663 10.1016/s0165-0173(98)00004-6

[17] Murer MG, Yan Q, Raisman-Vozari R. Brain-derived neurotrophic factor in the control human brain, and in Alzheimer's disease and Parkinson's disease. Progress in neurobiology. 2001;63(1):71–124. . 11040419 10.1016/s0301-0082(00)00014-9

[18] Davies P, Maloney AJ. Selective loss of central cholinergic neurons in Alzheimer's disease. Lancet. 1976;2(8000):1403 . 63862 10.1016/s0140-6736(76)91936-x

[19] Whitehouse PJ, Price DL, Struble RG, Clark AW, Coyle JT, Delon MR. Alzheimer's disease and senile dementia: loss of neurons in the basal forebrain. Science. 1982;215(4537):1237–9. . 7058341 10.1126/science.7058341

[20] Lu B, Nagappan G, Lu Y. BDNF and synaptic plasticity, cognitive function, and dysfunction. Handbook of experimental pharmacology. 2014;220:223–50. 10.1007/978-3-642-45106-5_9 . 24668475

[21] Laske C, Stellos K, Hoffmann N, Stransky E, Straten G, Eschweiler GW, et al Higher BDNF serum levels predict slower cognitive decline in Alzheimer's disease patients. The international journal of neuropsychopharmacology / official scientific journal of the Collegium Internationale Neuropsychopharmacologicum. 2011;14(3):399–404. 10.1017/S1461145710001008 . 20860877

[22] Typlt M, Mirkowski M, Azzopardi E, Ruettiger L, Ruth P, Schmid S. Mice with deficient BK channel function show impaired prepulse inhibition and spatial learning, but normal working and spatial reference memory. PloS one. 2013;8(11):e81270 10.1371/journal.pone.0081270 24303038 PMC3841135

[23] Yamada K, Tanaka T, Mamiya T, Shiotani T, Kameyama T, Nabeshima T. Improvement by nefiracetam of beta-amyloid-(1–42)-induced learning and memory impairments in rats. British journal of pharmacology. 1999;126(1):235–44. 10.1038/sj.bjp.0702309 10051141 PMC1565810

[24] Yamada K, Tanaka T, Han D, Senzaki K, Kameyama T, Nabeshima T. Protective effects of idebenone and alpha-tocopherol on beta-amyloid-(1–42)-induced learning and memory deficits in rats: implication of oxidative stress in beta-amyloid-induced neurotoxicity in vivo. The European journal of neuroscience. 1999;11(1):83–90. . 9987013 10.1046/j.1460-9568.1999.00408.x

[25] Nakamura S, Murayama N, Noshita T, Annoura H, Ohno T. Progressive brain dysfunction following intracerebroventricular infusion of beta(1–42)-amyloid peptide. Brain research. 2001;912(2):128–36. . 11532428 10.1016/s0006-8993(01)02704-4

[26] Wang Y, Liu J, Zhang Z, Bi P, Qi Z, Zhang C. Anti-neuroinflammation effect of ginsenoside Rbl in a rat model of Alzheimer disease. Neuroscience letters. 2011;487(1):70–2. 10.1016/j.neulet.2010.09.076 . 20933058

[27] Zhu X, Chen C, Ye D, Guan D, Ye L, Jin J, et al Diammonium glycyrrhizinate upregulates PGC-1alpha and protects against Abeta1-42-induced neurotoxicity. PloS one. 2012;7(4):e35823 10.1371/journal.pone.0035823 22540007 PMC3335163

[28] Narisawa-Saito M, Wakabayashi K, Tsuji S, Takahashi H, Nawa H. Regional specificity of alterations in NGF, BDNF and NT-3 levels in Alzheimer's disease. Neuroreport. 1996;7(18):2925–8. . 9116211 10.1097/00001756-199611250-00024

[29] Conner JM, Lauterborn JC, Yan Q, Gall CM, Varon S. Distribution of brain-derived neurotrophic factor (BDNF) protein and mRNA in the normal adult rat CNS: evidence for anterograde axonal transport. The Journal of neuroscience: the official journal of the Society for Neuroscience. 1997;17(7):2295–313. . 9065491 10.1523/JNEUROSCI.17-07-02295.1997 PMC6573520

[30] Ferrer I, Marin C, Rey MJ, Ribalta T, Goutan E, Blanco R, et al BDNF and full-length and truncated TrkB expression in Alzheimer disease. Implications in therapeutic strategies. Journal of neuropathology and experimental neurology. 1999;58(7):729–39. . 10411343 10.1097/00005072-199907000-00007

[31] Hock C, Heese K, Hulette C, Rosenberg C, Otten U. Region-specific neurotrophin imbalances in Alzheimer disease: decreased levels of brain-derived neurotrophic factor and increased levels of nerve growth factor in hippocampus and cortical areas. Archives of neurology. 2000;57(6):846–51. . 10867782 10.1001/archneur.57.6.846

[32] Lee J, Fukumoto H, Orne J, Klucken J, Raju S, Vanderburg CR, et al Decreased levels of BDNF protein in Alzheimer temporal cortex are independent of BDNF polymorphisms. Experimental neurology. 2005;194(1):91–6. 10.1016/j.expneurol.2005.01.026 . 15899246

[33] Peng S, Wuu J, Mufson EJ, Fahnestock M. Precursor form of brain-derived neurotrophic factor and mature brain-derived neurotrophic factor are decreased in the pre-clinical stages of Alzheimer's disease. Journal of neurochemistry. 2005;93(6):1412–21. 10.1111/j.1471-4159.2005.03135.x . 15935057

[34] Laske C, Stransky E, Leyhe T, Eschweiler GW, Maetzler W, Wittorf A, et al BDNF serum and CSF concentrations in Alzheimer's disease, normal pressure hydrocephalus and healthy controls. Journal of psychiatric research. 2007;41(5):387–94. 10.1016/j.jpsychires.2006.01.014 . 16554070

[35] Fischer W, Sirevaag A, Wiegand SJ, Lindsay RM, Bjorklund A. Reversal of spatial memory impairments in aged rats by nerve growth factor and neurotrophins 3 and 4/5 but not by brain-derived neurotrophic factor. Proceedings of the National Academy of Sciences of the United States of America. 1994;91(18):8607–11. 8078930 10.1073/pnas.91.18.8607 PMC44655

[36] Pelleymounter MA, Cullen MJ, Baker MB, Gollub M, Wellman C. The effects of intrahippocampal BDNF and NGF on spatial learning in aged Long Evans rats. Molecular and chemical neuropathology / sponsored by the International Society for Neurochemistry and the World Federation of Neurology and research groups on neurochemistry and cerebrospinal fluid. 1996;29(2–3):211–26. 10.1007/BF02815003 . 8971697

[37] Croll SD, Suri C, Compton DL, Simmons MV, Yancopoulos GD, Lindsay RM, et al Brain-derived neurotrophic factor transgenic mice exhibit passive avoidance deficits, increased seizure severity and in vitro hyperexcitability in the hippocampus and entorhinal cortex. Neuroscience. 1999;93(4):1491–506. 10501474 10.1016/s0306-4522(99)00296-1 PMC2504500

[38] Kang H, Schuman EM. Long-lasting neurotrophin-induced enhancement of synaptic transmission in the adult hippocampus. Science. 1995;267(5204):1658–62. . 7886457 10.1126/science.7886457

[39] Sweatt JD. Mitogen-activated protein kinases in synaptic plasticity and memory. Current opinion in neurobiology. 2004;14(3):311–7. 10.1016/j.conb.2004.04.001 . 15194111

[40] Webster B, Hansen L, Adame A, Crews L, Torrance M, Thal L, et al Astroglial activation of extracellular-regulated kinase in early stages of Alzheimer disease. Journal of neuropathology and experimental neurology. 2006;65(2):142–51. 10.1097/01.jnen.0000199599.63204.6f . 16462205

[41] Dineley KT, Westerman M, Bui D, Bell K, Ashe KH, Sweatt JD. Beta-amyloid activates the mitogen-activated protein kinase cascade via hippocampal alpha7 nicotinic acetylcholine receptors: In vitro and in vivo mechanisms related to Alzheimer's disease. The Journal of neuroscience: the official journal of the Society for Neuroscience. 2001;21(12):4125–33. . 11404397 10.1523/JNEUROSCI.21-12-04125.2001 PMC6762764

[42] Amadoro G, Ciotti MT, Costanzi M, Cestari V, Calissano P, Canu N. NMDA receptor mediates tau-induced neurotoxicity by calpain and ERK/MAPK activation. Proceedings of the National Academy of Sciences of the United States of America. 2006;103(8):2892–7. 10.1073/pnas.0511065103 16477009 PMC1413822

[43] Hyman BT, Elvhage TE, Reiter J. Extracellular signal regulated kinases. Localization of protein and mRNA in the human hippocampal formation in Alzheimer's disease. The American journal of pathology. 1994;144(3):565–72. 8129042 PMC1887090

[44] Zhang L, Xu T, Wang S, Yu L, Liu D, Zhan R, et al Curcumin produces antidepressant effects via activating MAPK/ERK-dependent brain-derived neurotrophic factor expression in the amygdala of mice. Behavioural brain research. 2012;235(1):67–72. 10.1016/j.bbr.2012.07.019 . 22820234

[45] Wang R, Li YH, Xu Y, Li YB, Wu HL, Guo H, et al Curcumin produces neuroprotective effects via activating brain-derived neurotrophic factor/TrkB-dependent MAPK and PI-3K cascades in rodent cortical neurons. Progress in neuro-psychopharmacology & biological psychiatry. 2010;34(1):147–53. 10.1016/j.pnpbp.2009.10.016 . 19879308

[46] Tong L, Balazs R, Thornton PL, Cotman CW. Beta-amyloid peptide at sublethal concentrations downregulates brain-derived neurotrophic factor functions in cultured cortical neurons. The Journal of neuroscience: the official journal of the Society for Neuroscience. 2004;24(30):6799–809. 10.1523/JNEUROSCI.5463-03.2004 . 15282285 PMC6729714

[47] Xu X, Zhan M, Duan W, Prabhu V, Brenneman R, Wood W, et al Gene expression atlas of the mouse central nervous system: impact and interactions of age, energy intake and gender. Genome biology. 2007;8(11):R234 10.1186/gb-2007-8-11-r234 17988385 PMC2258177

[48] Llorens-Martin M, Jurado J, Hernandez F, Avila J. GSK-3beta, a pivotal kinase in Alzheimer disease. Frontiers in molecular neuroscience. 2014;7:46 10.3389/fnmol.2014.00046 24904272 PMC4033045

[49] Cross DA, Alessi DR, Cohen P, Andjelkovich M, Hemmings BA. Inhibition of glycogen synthase kinase-3 by insulin mediated by protein kinase B. Nature. 1995;378(6559):785–9. 10.1038/378785a0 . 8524413

[50] DaRocha-Souto B, Coma M, Perez-Nievas BG, Scotton TC, Siao M, Sanchez-Ferrer P, et al Activation of glycogen synthase kinase-3 beta mediates beta-amyloid induced neuritic damage in Alzheimer's disease. Neurobiology of disease. 2012;45(1):425–37. 10.1016/j.nbd.2011.09.002 21945540 PMC3694284

