# Supplementary figures and images for: Curcumin Improves Amyloid β-Peptide (1-42) Induced Spatial Memory Deficits through BDNF-ERK Signaling Pathway
Source: PLoS One. 2015 Jun 26;10(6):e0131525. doi: 10.1371/journal.pone.0131525 (PMC4482657; doi:10.1371/journal.pone.0131525)

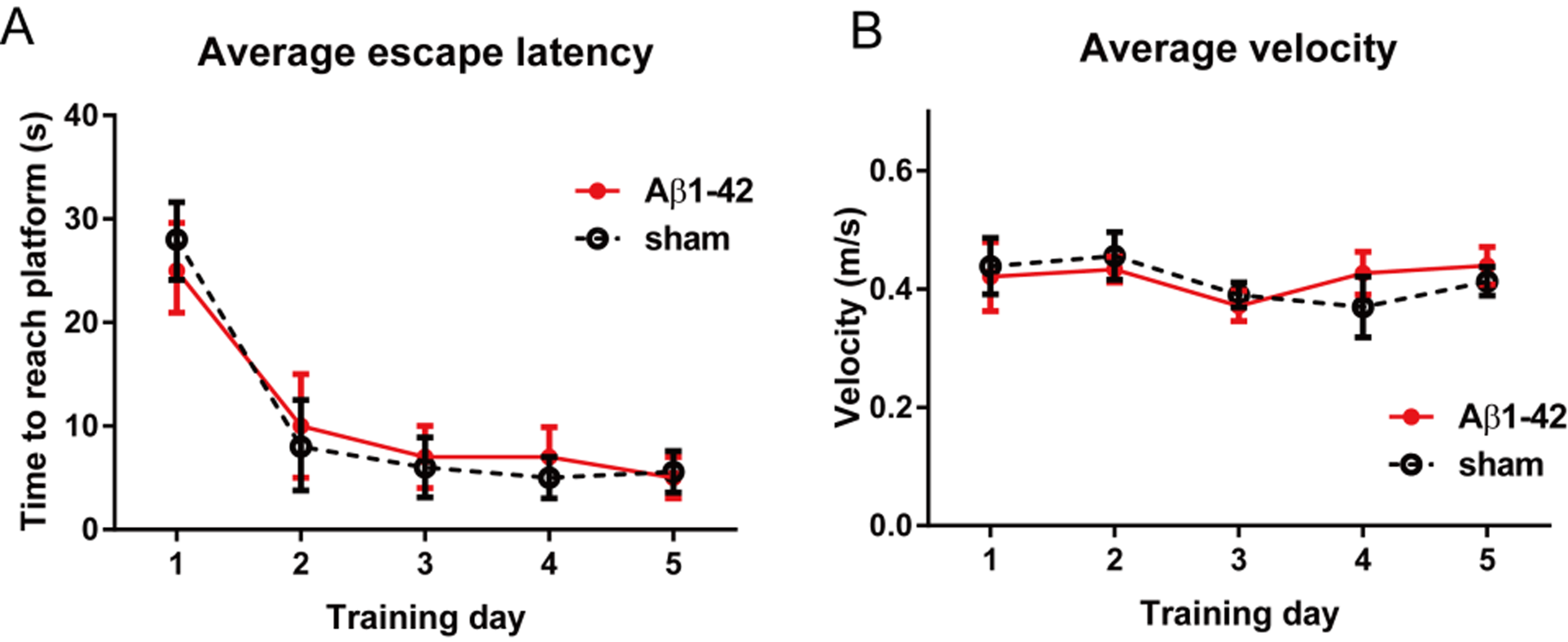

Supplement: S1 Fig — Performance in the Morris water maze using cued platform training was assessed by (A) escape latency, (B) swim velocity during 5 training days. Each point represents the mean ± SEM for the two trials of each group on each day. n = 6/group. (TIF) [file pone.0131525.s001.tif]

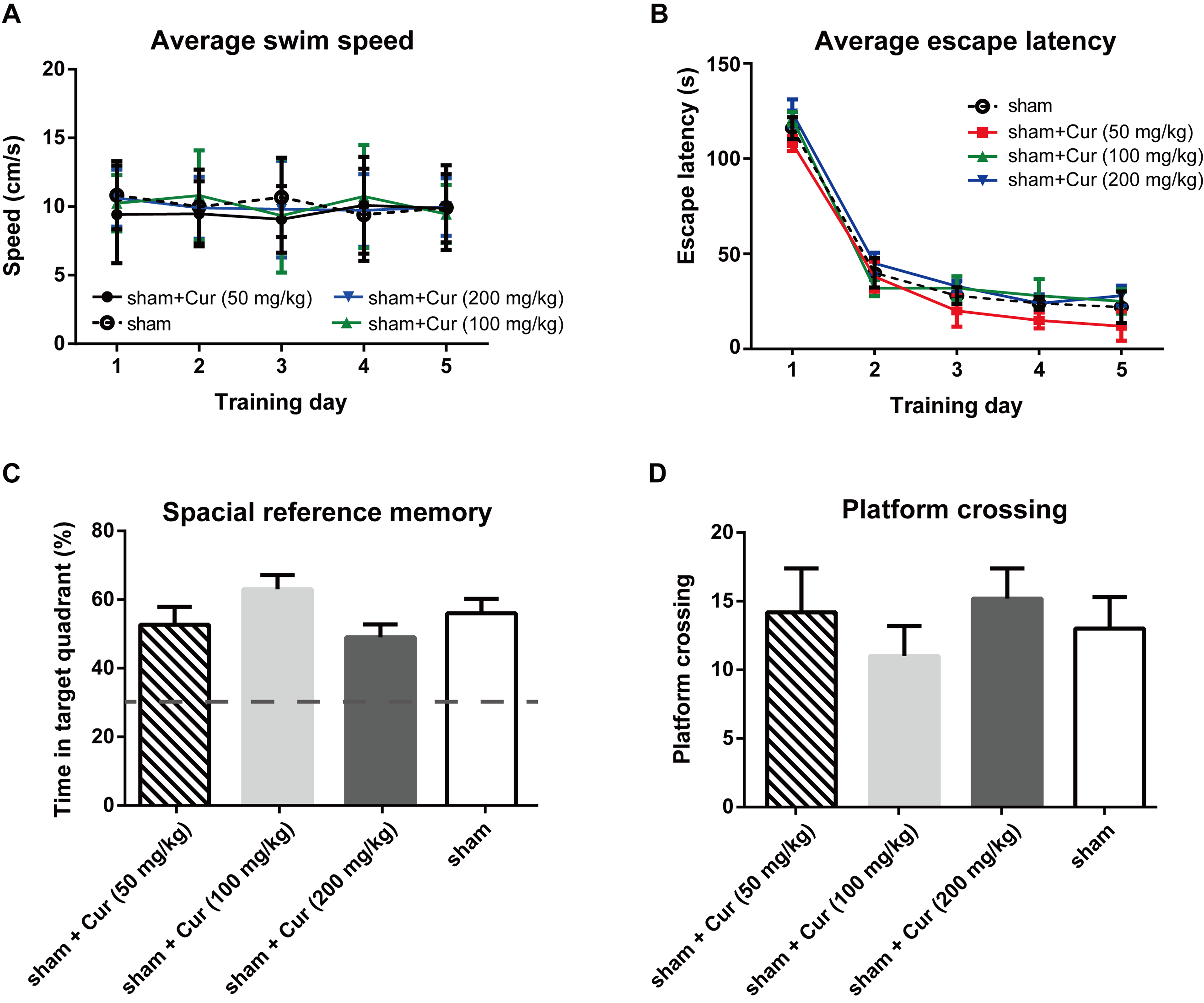

Supplement: S2 Fig — Curcumin (50, 100 and 200 mg/kg) was administrated i.p. for 5 consecutive days (Day 6–10) in rats without Aβ injection (ie. the sham+Cur group). (A) Swim speed in each training trial. (B) The escape latency during the water maze training trials. (C) The time spent in the target quadrant and (D) the number of times crossing the platform in the probe task. n = 6/group. (TIF) [file pone.0131525.s002.tif]

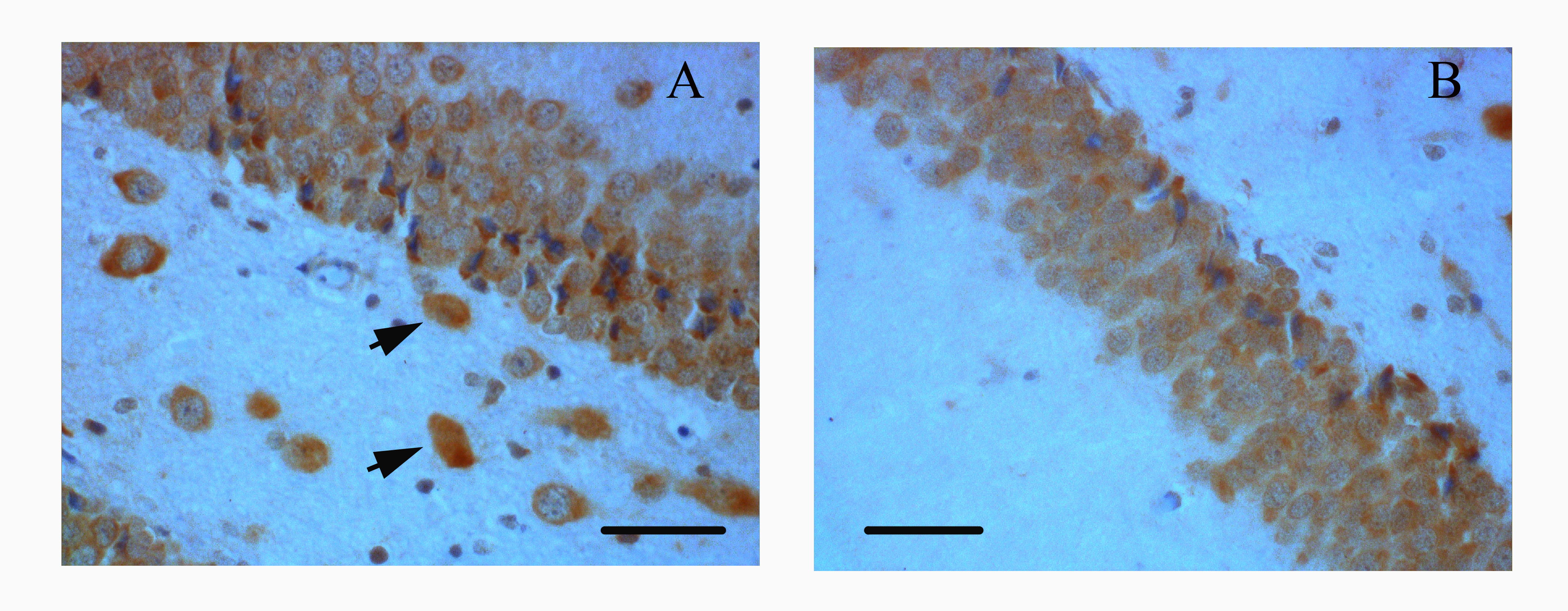

Supplement: S3 Fig — Representative figures of Aβ1-42 immunohistochemistry from model (A) and control groups (B), respectively. Scale bar 20 μm. The black arrows are showing the positive cells from immunostaining. (TIF) [file pone.0131525.s003.tif]
